# Efficacy of echocardiography for differential diagnosis of left ventricular hypertrophy: special focus on speckle-tracking longitudinal strain

**DOI:** 10.1007/s12574-020-00508-3

**Published:** 2021-01-18

**Authors:** Hidekazu Tanaka

**Affiliations:** Division of Cardiovascular Medicine, Department of Internal Medicine, Kobe University Graduate School of Medicine, 7-5-2, Kusunoki-cho, Chuo-ku, Kobe, 650-0017 Japan

**Keywords:** Left ventricular hypertrophy, Speckle-tracking strain, Global longitudinal strain, Echocardiography

## Abstract

Left ventricular (LV) hypertrophy (LVH) is a frequent imaging finding in daily clinical practice, and its presence is associated with poor outcomes and ventricular arrhythmias. It is commonly detected in athletes, arterial hypertension, aortic stenosis, hypertrophic cardiomyopathy, cardiac amyloidosis, Fabry disease, or Friedreich’s ataxia. Echocardiography plays an important role in detecting LVH and underlying causes in current clinical practice. While echocardiography is essential for the quantification and early detection of LV structural findings for various cardiovascular diseases, it has been reported that speckle-tracking echocardiographic parameters are also useful for the detection of early LV structural abnormalities. In particular, global longitudinal strain (GLS) assessed by two-dimensional speckle-tracking echocardiography is reportedly a sensitive marker for early subtle abnormalities of LV myocardial performance, helpful for the prediction of outcomes for various cardiac diseases, and superior to conventional echocardiographic indices. GLS is determined as the averaged peak longitudinal strain of 18 LV segments from standard apical views and can be assessed as a polar plot. This polar plot longitudinal strain mapping offers an intuitive visual overview of the global and regional LV longitudinal myocardial function status of various cardiomyopathies with LVH. This mapping is clinically practicable and the plot patterns obtainable as the result of further development of this technique for clinical practice provide clues to the etiology of cardiomyopathies. This article reviews the efficacy of echocardiography for differential diagnosis of LVH, with a special focus on the utility of speckle-tracking longitudinal strain.

## Introduction

Left ventricular (LV) hypertrophy (LVH) is a frequent imaging finding in daily clinical practice, and its presence is associated with poor outcomes and ventricular arrhythmias [1, 2]. It is commonly detected in athletes following long-term exercise training, in patients with arterial hypertension and aortic stenosis (AS) due to persistent pressure overload, in those with hypertrophic cardiomyopathy (HCM), as well as those with systemic diseases such as cardiac amyloidosis, Fabry disease, and Friedreich’s ataxia. Echocardiography plays an important role in the detection of LVH and underlying causes in current clinical practice [3, 4]. While echocardiography is essential for the quantification and early detection of LV structural findings for various cardiovascular diseases [5], it has been reported that speckle-tracking echocardiographic parameters are also useful for the detection of early LV structural abnormalities. In particular, global longitudinal strain (GLS) assessed by two-dimensional speckle-tracking echocardiography is reportedly a sensitive marker for early subtle abnormalities of LV myocardial performance, helpful for the prediction of outcomes for various cardiac diseases including cardiomyopathies, and superior to conventional echocardiographic indices [6–9]. GLS is determined as the averaged peak longitudinal strain of 18 LV segments from the three standard apical views and can be assessed as a polar plot by ordinary. The polar plot longitudinal strain mapping offers an intuitive visual overview of the global and regional LV longitudinal myocardial function status in various cardiomyopathies with LVH. The polar plot longitudinal strain mapping is clinically practicable and the plot patterns obtainable as the result of further development of this technique in clinical practice provide clues to the etiology of cardiomyopathies, especially for patients with preserved LV ejection fraction (LVEF).

This article reviews the efficacy of echocardiography for differential diagnosis of LVH caused by arterial hypertension, HCM, cardiac amyloidosis, Fabry disease, AS, athlete’s heart, and Friedreich’s ataxia, with a special focus on the utility of speckle-tracking longitudinal strain. In this review article, the value of GLS is expressed as an absolute value.

## Arterial hypertension

Cardiovascular diseases are common in the general population, and arterial hypertension is one of the most important risk factors for such diseases. LVH is a relatively early manifestation and a common finding in patients with arterial hypertension. As a consequence of arterial hypertension, it is associated with an increased risk of major cardiovascular events including heart failure, coronary heart disease, stroke, and sudden death [1, 10, 11]. Arterial hypertension is one of the most common causes of LVH, although the majority of patients with hypertensive LVH have a maximal interventricular septal thickness of < 15 mm [12].

**Fig. 1:**
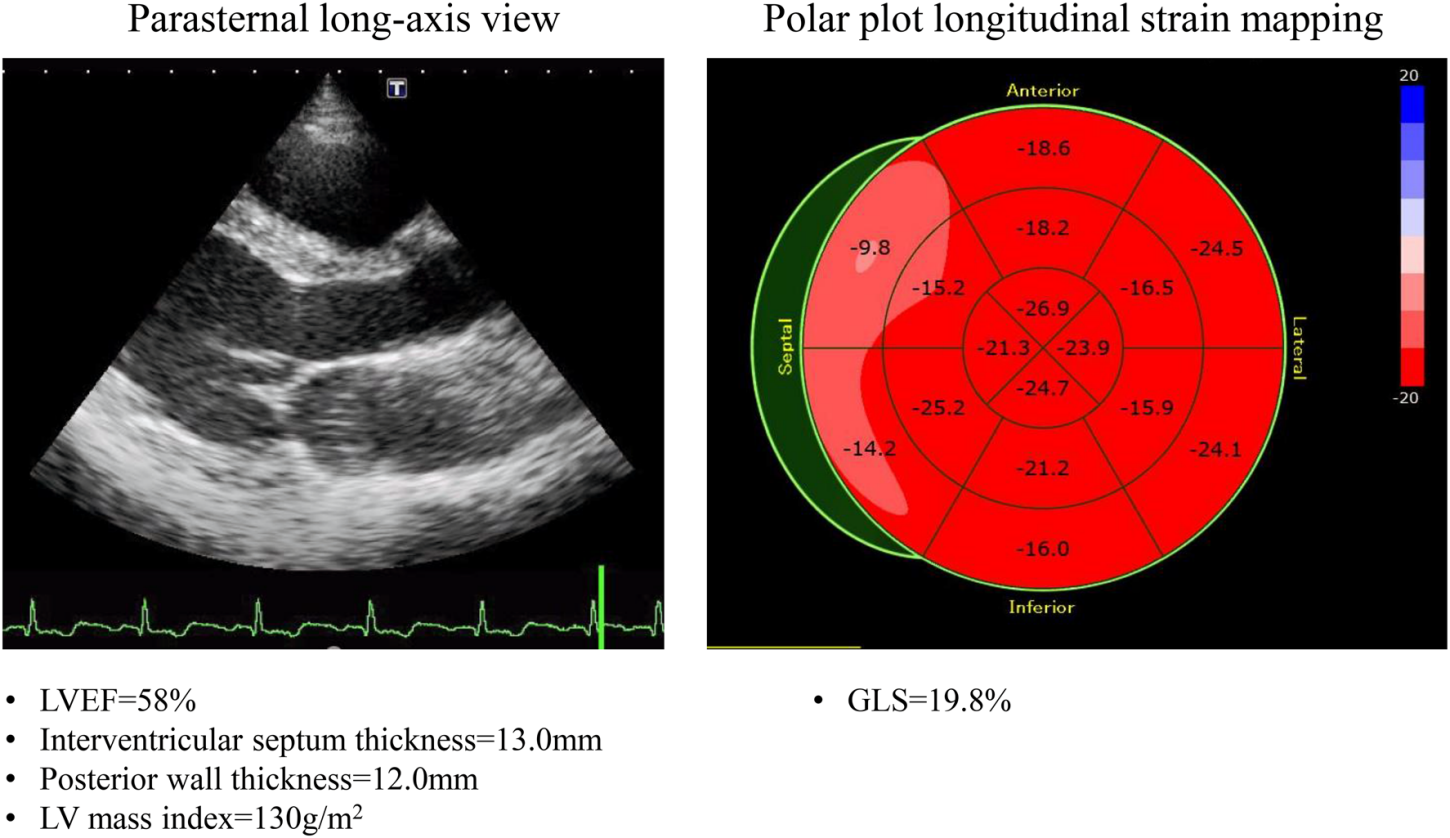
Examples of the parasternal long-axis view and the longitudinal strain bull’s eye plot for a 71-year-old female with arterial hypertension. GLS of 19.8% is normal, but the longitudinal strain in the basal septum is reduced compared to that in other segments

GLS helps to unmask early subclinical LV systolic dysfunction in patients with arterial hypertension. The basal septum is the first segment to undergo changes under the influence of pressure overload, and GLS is further reduced at this site (Fig. 1) [13]. It was previously reported that 46% of patients showed low GLS values (< 17%), and low GLS was associated with long-lasting arterial hypertension and uncontrolled blood pressure for 200 outpatients with arterial hypertension with preserved LVEF [14]. Moreover, the degree of GLS is strongly associated with LV diastolic function, independent of changes in afterload and degree of LVH [15].

## Hypertrophic cardiomyopathy

HCM is the most frequent genetically determined cardiomyopathy in adults and is characterized by non-symmetric LVH in the absence of other cardiovascular or systemic diseases. Characteristic echocardiographic findings for HCM include asymmetrical septal hypertrophy and systolic anterior motion of the mitral valve. Typically, LV end-diastolic wall thickness ≥ 15 mm is often observed in one or more LV myocardial segments, but isolated apical and other atypical distributions have also been reported. In cases with less wall thickening (13–14 mm), the diagnosis of HCM is often challenging.

**Fig. 2:**
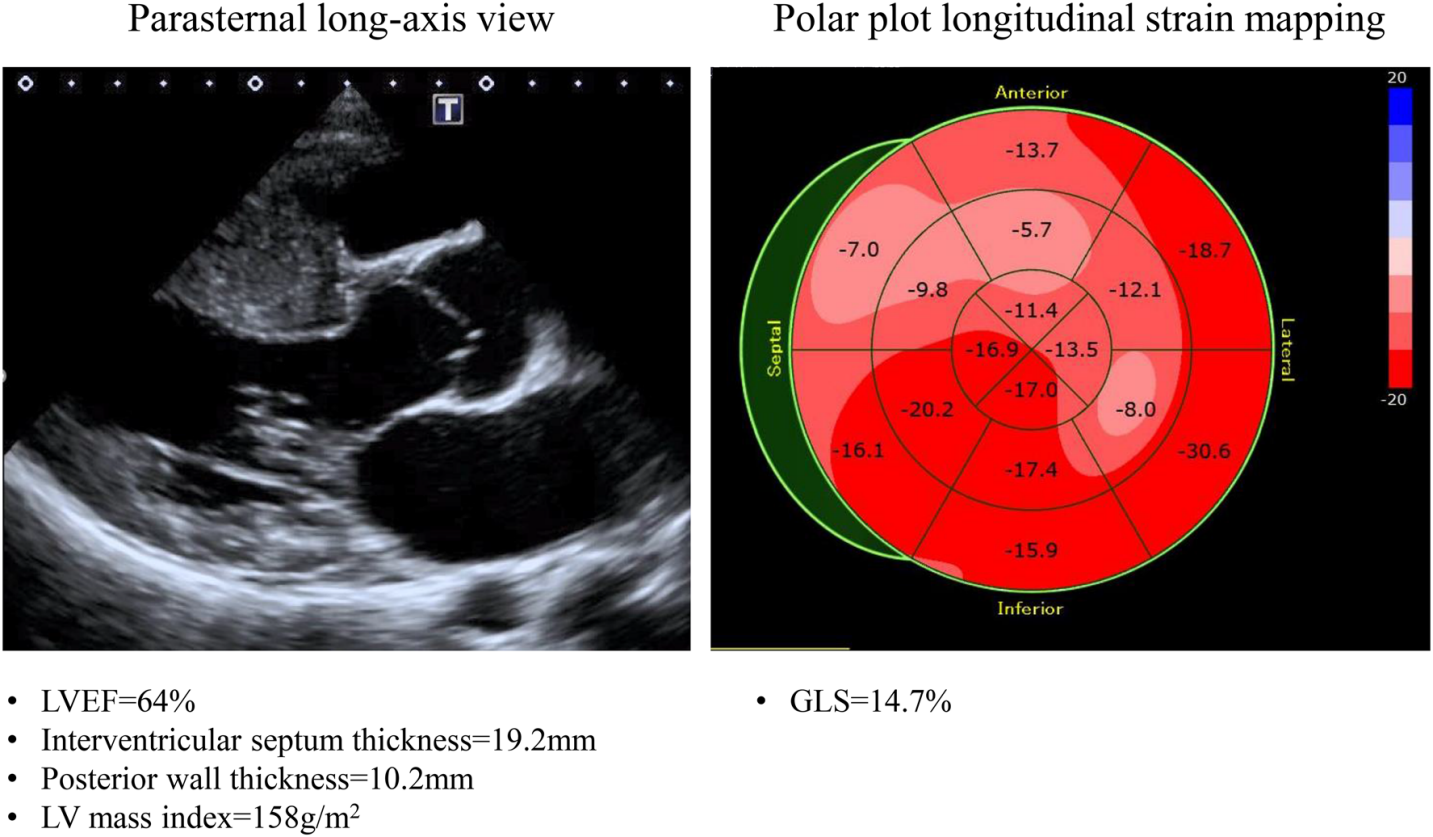
Examples of the parasternal long-axis view and the longitudinal strain bull’s eye plot for a 48-year-old male with HCM. GLS of 14.7% is low, and longitudinal strain in the region of the interventricular septum with its increased wall thickness is further reduced

It has been reported that LV longitudinal myocardial function is reduced even though LVEF is normal and may become abnormal before wall thickness starts to increase [16]. LV longitudinal myocardial function is typically reduced at the site of hypertrophy, especially in the region of the interventricular septum (Fig. 2) [17]. Patients with HCM were found to have similar LVEF but worse GLS compared to healthy individuals, and those with ventricular arrhythmias showed worse GLS than those without them. Therefore, GLS is an appropriate parameter for evaluating LV systolic function in HCM and may improve risk stratification of ventricular arrhythmia for such patients [18]. Moreover, abnormal GLS was found to be a better predictor of outcome for HCM than conventional echocardiographic parameters, and reduced GLS to be an independent parameter associated with poor cardiac outcomes [19].

## Cardiac amyloidosis

Amyloidosis is a multi-systemic disease characterized by the deposition of amyloid fibrosis in the intercellular space of various organs [20]. Cardiac involvement occurs in up to 50% of patients with primary amyloidosis and indicates almost invariably a grave prognosis. Conventional echocardiographic features associated with cardiac amyloidosis include concentric LVH and right ventricular hypertrophy, normal LV cavity size, dilated atria, and pericardial effusion. LV diastolic abnormalities are generally recognized as the earliest manifestation of cardiac amyloidosis [21], while LV global systolic function remains normal until the later stages of the disease [22]. The myocardial texture often features a distinct “granular sparkling” appearance [23], and this echocardiographic feature is well known as a key factor in the diagnosis of cardiac amyloidosis. However, this can occur in other causes of LVH, and although high specificity rates are quoted (71–81%), the populations studied were those referred with suspected amyloid fibrosis, so that this specificity may not be reflective of real-life practice. Moreover, sensitivity for the “granular sparkling” appearance tends to be low, with this pattern seen in only 26–36% of cardiac amyloidosis cases [24]. It should be noted that this granular pattern applies only to standard echocardiographic imaging, without the inclusion of tissue harmonics, as this increases myocardial echogenicity in general. Newer echocardiographic image processing techniques may also reduce granular appearance. Thus, although increased echogenicity is common in cardiac amyloidosis, its usefulness as a discriminating factor is limited.

**Fig. 3:**
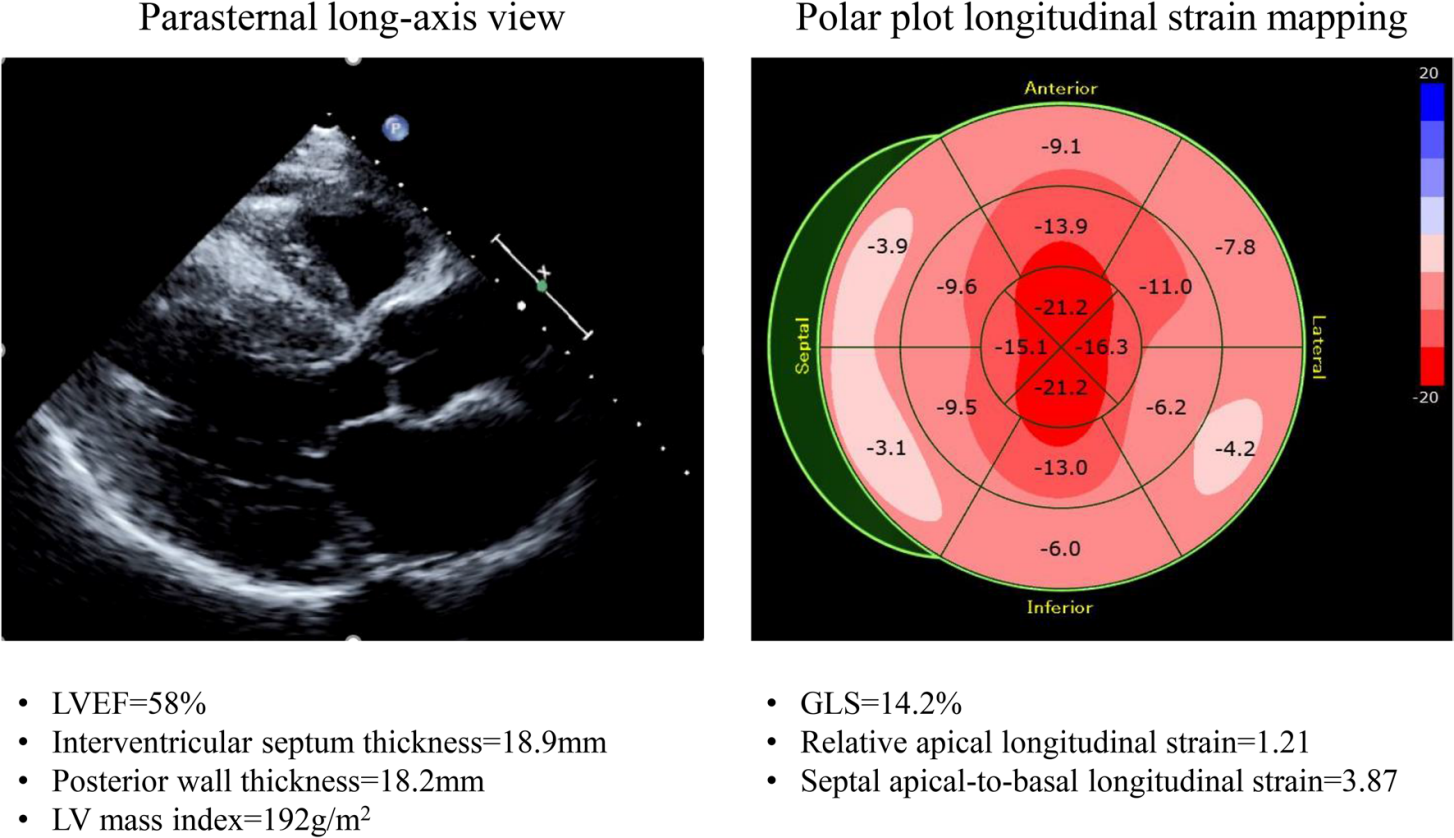
Examples of the parasternal long-axis view and the longitudinal strain bull’s eye plot for a 72-year-old male with variant transthyretin cardiac amyloidosis. GLS of 14.2% is low, while an apical sparing pattern can be observed

It is noteworthy that cardiac amyloidosis is characterized by regional variations in longitudinal strain from base to apex. A longitudinal strain gradient with preserved systolic strain at apical segments and significantly reduced systolic strain at mid and basal segments is consistently observed [25, 26]. Previous studies have demonstrated that this pattern, known as “Apical Sparing”, is specific, thus suited to differentiate patients with cardiac amyloidosis from patients with other causes of LVH [26, 27]. Phelan et al. compared 55 consecutive patients with cardiac amyloidosis with 30 control patients with LVH comprising 15 patients with HCM and 15 with AS [25]. A relative apical longitudinal strain of 1.0, determined by using the equation [average apical longitudinal strain/(average basal-longitudinal strain + mid-longitudinal strain)], was associated with a sensitivity of 93% and specificity of 82% for differentiating patients with cardiac amyloidosis from control patients with LVH with an area under the curve of 0.94. Moreover, Liu et al. showed that a ratio of a septal apical-to-basal segmental longitudinal strain of > 2.1 can differentiate cardiac amyloidosis from other causes of concentric LVH with a sensitivity of 88%, specificity of 85%, positive predictive value of 67%, and negative predictive value of 96% [27]. This specific relative apical sparing can be easily visualized by polar plot longitudinal strain mapping for patients with cardiac amyloidosis (Fig. 3). Apical sparing is observed both in patients with transthyretin cardiac amyloidosis and in those with amyloid light-chain amyloidosis. However, the strain value at the LV apex in patients with transthyretin cardiac amyloidosis has been shown to be lower than that in patients with amyloid light-chain cardiac amyloidosis [28]. Moreover, Barros-Gomes et al. showed that GLS predicted all-cause mortality and provide additional prognostic information for all-cause mortality better than established clinical, echocardiographic, and serological markers in 150 consecutive patients with amyloid light-chain cardiac amyloidosis and preserved LVEF [29]. They also used multivariate Cox regression analysis to show that GLS was an independent predictor of all-cause mortality. In addition, the Guideline for the Diagnosis and Treatment of Cardiac Amyloidosis provided by the Japanese Circulation Society mentions that speckle-tracking echocardiography is recommended for the differentiation of cardiac amyloidosis from other causes of LVH, and/or for the prediction of the prognosis as class I of recommendation and level B of evidence [30]. The speedier diagnosis of transthyretin cardiac amyloidosis by using apical sparing may therefore lead to earlier administration of tafamidis meglumine.

## Fabry disease

Fabry disease is an X-linked lysosomal storage disorder caused by α-galactosidase A deficiency. The clinical presentation is multisystemic, affecting the kidneys, heart, and nervous system. Cardiac pathophysiology is characterized by globotriaosylceramide accumulation within cardiomyocytes that cause LVH with subsequent myocardial replacement fibrosis. The most common cardiac manifestations include arrhythmias and heart failure, which are also responsible for the reduced life expectancy associated with Fabry disease [31]. Cardiomyopathy related to this disease is characterized by hypertrophy, increased LV volume, and gradual transformation to a more spherical LV shape, and myocardial replacement fibrosis. The use of the binary sign or endocardial stripe has been investigated as a hallmark of Fabry disease. The binary sign is the appearance of a bright, hyperechogenic region in the LV myocardium adjacent to a relatively low echo intensity region with a clear black and white interface. Pieroni et al. found a binary sign in 83% of patients with Fabry disease [32], but other studies have demonstrated a much lower prevalence of approximately 20% and have noted that the binary sign occurs more frequently in patients with LVH, which may partially account for this discrepancy [33, 34]. Thus, the diagnosis of Fabry disease by means of the presence of a binary sign is of limited utility.

**Fig. 4:**
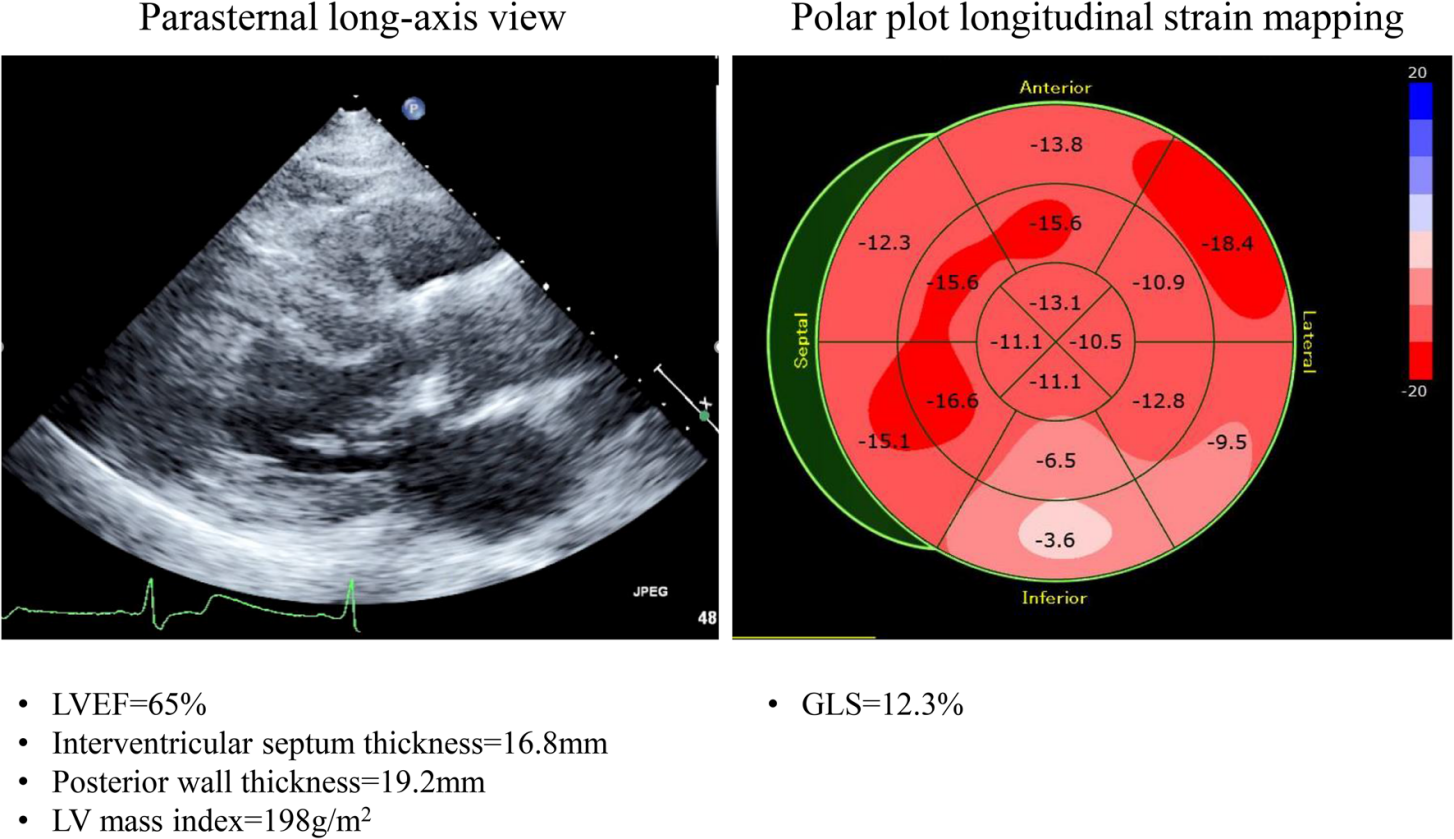
Examples of the parasternal long-axis view and the longitudinal strain bull’s eye plot for a 72-year-old female with Fabry disease. LVEF of 65% is preserved without wall motion abnormality, but GLS of 12.3% is low. Furthermore, longitudinal strain in the basal posterior-lateral wall was further reduced compared to that in other regions

The development of fibrosis is often localized in the basal posterior-lateral wall even with preserved LVEF [35]. The reduced longitudinal strain in the basal lateral wall was also found at the very early stages of Fabry disease in the absence of replacement fibrosis [31]. Furthermore, Kramer et al. found no significant difference in GLS between patients with normal and elevated sphericity index among 74 Fabry disease patients with preserved LVEF [36]. In contrast, longitudinal strain in the basal posterior-lateral wall, where replacement fibrosis is usually located, is significantly decreased in patients with an elevated sphericity index. Figure 4 shows examples of the parasternal long-axis view and the longitudinal strain bull’s eye plot for a 72-year-old female with Fabry disease. Her LVEF was preserved at 65% without wall motion abnormality, but GLS was as low as 12.3%. Furthermore, longitudinal strain in the basal posterior-lateral wall was further reduced compared to that in other regions.

## Aortic stenosis

AS is a growing health problem, and it should not be viewed as an isolated disease limited to the aortic valve, but as a systemic disease with increased peripheral vascular resistance caused by atherosclerosis and a concomitant deep alteration in LV structure, even in the presence of a preserved LVEF. LVEF is normal in most patients with AS even when symptoms develop, and valvular parameters, including transvalvular gradients and aortic valve area, are not useful for the prediction of clinical outcome after aortic valve replacement [37]. Lafitte et al. demonstrated that patients with severe AS and preserved LVEF had lower GLS compared with matched control subjects, and that this difference was more pronounced in the basal LV segments. A lower GLS was also found to be associated with a higher LV mass index and relative wall thickness, which supports the notion of a direct connection between concentric remodeling and contractile dysfunction [38]. From a physiological point of view, the pressure overload of AS triggers a continuum of changes that start from myocyte hypertrophy and interstitial reactive fibrosis, leading to a self-perpetuating process of cellular atrophy, myocyte death, and replacement fibrosis, eventually causing a progressive deterioration of myocardial function with poor prognosis [39].

**Fig. 5:**
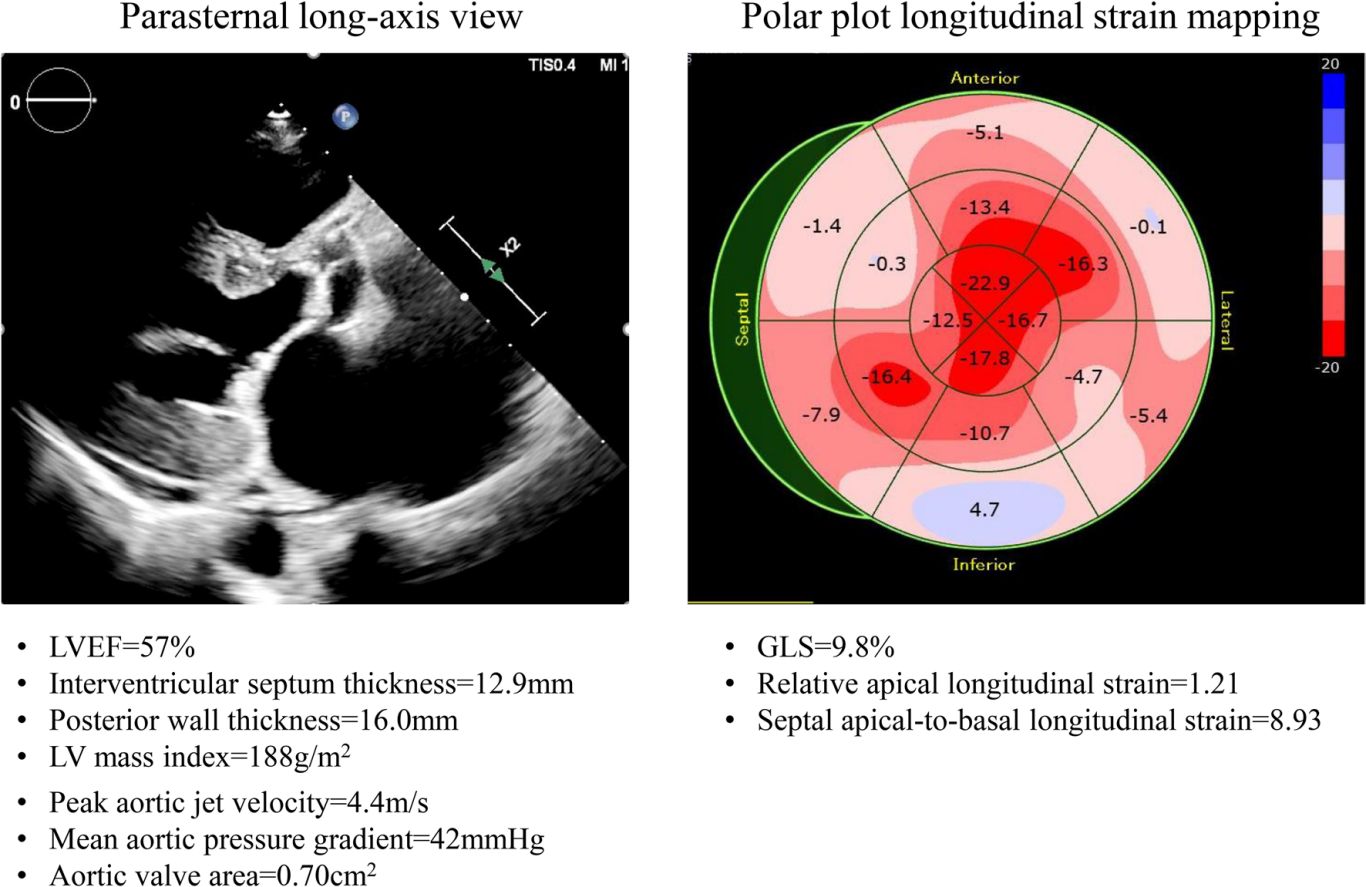
Examples of the parasternal long-axis view and the longitudinal strain bull’s eye plot for an 82-year-old male with severe AS who was referred for transcatheter aortic valve implantation. GLS of 9.8% is low, but an apical sparing pattern can be observed. Moreover, the diagnosis of this patient was complicated by wild-type transthyretin cardiac amyloidosis

AS combined with transthyretin cardiac amyloidosis has attracted attention in recent years, because their coexistence must always be taken into consideration since both transthyretin cardiac amyloidosis and severe AS result in LVH, thus interfering with the detection of suspected coexistence of transthyretin cardiac amyloidosis. Treibel et al. reported that the prevalence of wild-type transthyretin cardiac amyloid was 6% among patients with severe AS aged > 65 years undergoing surgical aortic valve replacement, and was associated with a poor outcome [40]. In addition, Castaño et al. reported that transthyretin cardiac amyloidosis was prevalent in 16% of patients with severe AS undergoing transcatheter aortic valve implantation and was associated with a severe low-flow low-gradient AS phenotype despite only mildly reduced LVEF [41]. Another study observed occult cardiac amyloidosis in 13.9% of severe aortic stenosis patients prior to transcatheter aortic valve implantation [42]. Thus, the presence of transthyretin cardiac amyloidosis can be suspected based on various red flags, especially apical sparing, in severe AS patients who have been referred for transcatheter aortic valve implantation or surgical aortic valve replacement. This detection procedure has become especially significant since tafamidis meglumine was launched for the treatment of transthyretin cardiac amyloidosis (Fig. 5).

## Athlete’s heart

Physiological hypertrophy can be detected in the heart of athletes, whose LV dilation and LVH may be pronounced enough to mimic a pathological state, but LV systolic and diastolic function is normal or even supranormal. In top-level athletes, LV end-diastolic diameter is often increased, although LVEF is normal, with normal or supranormal stroke volume and systolic peak velocity > 9 cm/s by means of tissue Doppler imaging [43]. Some athletes may present enhanced early diastolic LV filling [44], so that assessment of LV diastolic function might be a key factor for differentiating physiological LVH due to adaptation to exercise from pathological LVH [45]. Identification of cardiomyopathies can be challenging when the wall thickness is between 12 and 16 mm (the so-called gray zone of LVH). Kansal et al. reported the utility of conventional echocardiographic and speckle-tracking parameters for distinguishing the pathologic LVH of hypertrophic cardiomyopathy from the physiologic LVH of professional football players when septal wall thickness falls within a "gray zone" between 12 and 16 mm [46]. They studied 28 professional football players and 21 patients with HCM, with septal wall thicknesses of 12–16 mm, along with 17 normal controls. They showed that relative wall thickness and GLS of patients with HCM were significantly larger than those of professional football players. They also showed that relative wall thickness most accurately differentiated professional football players from patients with HCM. Afonso et al. also compared GLS of 56 patients with HCM, 34 patients with LVH due to arterial hypertension, 27 professional athletes with LVH and 12 healthy controls in sinus rhythm with preserved LVEF [47]. They showed that GLS of professional athletes with LVH (17.8 ± 2.2%) was mildly reduced compared to healthy controls (18.7 ± 1.8%), and was similar to that of patients with LVH due to arterial hypertension (17.8 ± 3.1%), but significantly higher compared to that of patients with HCM (11.2 ± 4.2%).

## Friedreich’s ataxia

Friedreich’s ataxia is an autosomal recessive neurodegenerative disease caused by a guanine-adenine-adenine triplet repeat expansion in the first intron of frataxin [50]. The intronic expansion leads to a specific iron-sulfur protein deficiency, resulting in intra-mitochondrial iron accumulation. Besides the neurologic manifestation, cardiac involvement and endocrine involvement are also frequently observed [27]. A concentric LVH with an end-diastolic wall thickness of less than 15 mm is the usual echocardiographic feature [48]. Around 40% of patients with Friedreich’s ataxia show concentric remodeling, 35% concentric hypertrophy, and only 5% eccentric hypertrophy [49]. Global LV systolic and diastolic function remain normal in most patients with Friedreich’s ataxia, and only end-stage patients with this disease develop reduced LVEF with global hypokinesia and slightly dilated LV chamber[3]. Electrocardiographic abnormalities are often the earliest sign of Friedreich’s ataxia. At this early stage, echocardiography results are usually normal and the polar plot longitudinal strain mapping shows a similar pattern as that of healthy subjects. In patients with Friedreich’s ataxia with concentric LVH and normal LVEF, the polar plot longitudinal strain mapping pattern indicates a mildly reduced GLS [50]. Myocardial fibrosis develops gradually, leading to LV wall thinning and LV dilatation during the disease progression, while LVEF remains preserved for a long time until the end-stage of the disease [48]. Noteworthy is that the LV wall thinning appears to be diffuse in patients with Friedreich’s ataxia, which is different from the typical findings for Fabry cardiomyopathy. The polar plot longitudinal strain mapping shows significantly reduced GLS when LVEF is reduced. Additionally, Friedreich’s ataxia shares some echocardiographic features with cardiac amyloidosis in terms of morphology, including concentric LVH with a sparkling granular texture of myocardium. Different from cardiac amyloidosis, though, LV diastolic function of patients with Friedreich’s ataxia can be normal or only mildly impaired. Moreover, a longitudinal base-to-apex strain gradient, which is frequently evidenced in cardiac amyloidosis, is rarely detected in patients with Friedreich’s ataxia [27]. Furthermore, the underlying mechanisms of myocardial dysfunction in patients with Friedreich’s ataxia might be associated with myocyte cellular hypertrophy, iron deposits, focal necrosis, and diffuse fibrosis [51].

## Conclusion

Detection of a disease-related typical deformation pattern and polar plot longitudinal strain mapping may provide valuable clues for the final diagnosis in some patients with unclear LVH (Table 1).

**Table 1:** Strain pattern in different models of LVH

Type of LVH	GLS value	Typical impairment of longitudinal strain
Arterial hypertension	Normal or reduced	Basal septum
Hypertrophic cardiomyopathy	Reduced	The site of hypertrophy, especially in the region of the interventricular septum
Cardiac amyloidosis	Reduced	Regional variations in longitudinal strain from base-to-apex (Apical sparing)
Fabry disease	Reduced	Basal posterior-lateral wall
Aortic stenosis	Reduced	Basal LV segments
Athlete’s heart	Almost normal	None
Friedreich’s ataxia	Mildly reduced	None
